# Supplementary material for: A General Small-Angle X-ray Scattering-Based Screening Protocol for Studying Physical Stability of Protein Formulations
Source: Pharmaceutics. 2021 Dec 28;14(1):69. doi: 10.3390/pharmaceutics14010069 (PMC8778066; doi:10.3390/pharmaceutics14010069)
Supplement: Supplementary file 1 [file pharmaceutics-14-00069-s001.zip › Supplementary data.pdf]

pharmaceutics

MDPI

Supplementary Materials: A General Small-Angle X-ray Scattering-Based Screening Protocol for Studying Physical Stability of Protein Formulations

Fangrong Zhang, Gesa Richter, Benjamin Bourgeois, Emil Spreitzer, Armin Moser, Andreas Keilbach, Petra Kotnik and Tobias Madl

	1	2	3	4	5	6	7	8	9	10	11	12
A	H_2O	empty	empty	empty	empty	empty	empty	empty	empty	empty	empty	empty
B	Citrate, pH 5.5	Malonate, pH 5.5	MES, pH 6.0	BIS-TRIS, pH 6.5	Phosphate, pH 7.0	Imidazole, pH 6.2	BIS-TRIS Propane, pH 7.0	MOPS, pH 8.0	TAPSO, pH 7.0	Tricine, pH 7.5	Bicine, pH 8.0	SPG, pH 5.5
C	Citrate, pH 6.0	Malonate, pH 6.0	MES, pH 6.5	BIS-TRIS, pH 7.0	Phosphate, pH 7.5	Imidazole, pH 6.5	BIS-TRIS Propane, pH 7.5	HEPES, pH 7.0	TAPSO, pH 7.5	Tricine, pH 8.0	Bicine, pH 8.5	SPG, pH 6.0
D	Citrate, pH 6.5	Malonate, pH 6.5	MES, pH 6.7	ADA, pH 6.0	Phosphate, pH 8.0	Imidazole, pH 7.0	BIS-TRIS Propane, pH 8.0	HEPES, pH 7.5	TAPSO, pH 8.0	Tricine, pH 8.5	TAPS, pH 7.7	SPG, pH 6.5
E	Citrate, pH 7.0	Malonate, pH 7.0	Maleate, pH 6.0	ADA, pH 6.5	PIPES, pH 6.1	Imidazole, pH 7.5	BIS-TRIS Propane, pH 8.5	HEPES, pH 8.0	TRIS, pH 7.1	Glycylglycine, pH 7.5	TAPS, pH 8.0	SPG, pH 7.0
F	Succinate, pH 5.5	Malonate, pH 7.5	Maleate, pH 6.5	ADA, pH 7.0	PIPES, pH 6.5	Imidazole, pH 7.8	MOPS, pH 6.5	DIPSO, pH 7.0	TRIS, pH 7.5	Glycylglycine, pH 8.0	TAPS, pH 8.5	SPG, pH 7.5
G	Succinate, pH 6.0	Malonate, pH 8.0	Maleate, pH 7.0	Phosphate, pH 6.0	PIPES, pH 7.0	BIS-TRIS Propane, pH 6.3	MOPS, pH 7.0	DIPSO, pH 7.5	TRIS, pH 8.0	Glycylglycine, pH 8.5	AMPD, pH 8.0	SPG, pH 8.0
H	Succinate, pH 6.5	MES, pH 5.5	BIS-TRIS, pH 6.0	Phosphate, pH 6.5	PIPES, pH 7.5	BIS-TRIS Propane, pH 6.5	MOPS, pH 7.5	DIPSO, pH 8.0	TRIS, pH 8.5	Bicine, pH 7.6	AMPD, pH 8.5	SPG, pH 8.5

Table S1. JBScreen Buffers contain common buffer stocks (0.5 M concentration) in the neutral pH range (5.5 - 8.5) in useful 0.5 pH unit increments. SPG buffer (column 12) is produced by mixing Succinic acid:Sodium dihydrogen phosphate:Glycine in the molar ratios 2:7:7. This 'Super Buffer' screens the pH range from 4.0 to 10.0 without changing the chemical composition of the buffer solution.

pharmaceutics MDPI

Supplementary Script

```
#!/bin/csh

rm *.csv
rm filenames.txt

dos2unix *.dat

foreach SAXSfile (`echo *.dat`)
#foreach SAXSfile (`echo RB1.dat`)

    sed '/^#/d'           $SAXSfile              > temp
    sed -e 's/,/ /g' temp                        > $SAXSfile.clean
    awk '{print $2}' $SAXSfile.clean > $SAXSfile.Int.txt
    echo -n                $SAXSfile             >> filenames.txt
    echo -n                ' '                   >> filenames.txt

end

    awk '{print $1}' $SAXSfile.clean > qrange.txt
    paste                  *.Int.txt             > SAXSCombined.txt

rm temp *.clean *.Int.txt *.clean

echo ""
echo "*************************************************************************"
echo "                              FINISHED                                    "
echo "*************************************************************************"
echo ""
```
